# Rule-Enhanced Active Learning for Semi-Automated Weak Supervision

**DOI:** 10.3390/ai3010013

**Published:** 2022-03-16

**Authors:** David Kartchner, Davi Nakajima An, Wendi Ren, Chao Zhang, Cassie S. Mitchell

**Affiliations:** 1 Laboratory for Pathology Dynamics, Georgia Institute of Technology and Emory University, Atlanta, GA 30332, USA;; 2 School of Computer Science, Georgia Institute of Technology, Atlanta, GA 30332, USA;; 3 Department of Biomedical Engineering, Georgia Institute of Technology and Emory University, Atlanta, GA 30332, USA; 4 Machine Learning Center at Georgia Tech, Georgia Institute of Technology, Atlanta, GA 30332, USA

**Keywords:** weak supervision, active learning, natural language processing, text classification, text mining, data labeling

## Abstract

A major bottleneck preventing the extension of deep learning systems to new domains is the prohibitive cost of acquiring sufficient training labels. Alternatives such as weak supervision, active learning, and fine-tuning of pretrained models reduce this burden but require substantial human input to select a highly informative subset of instances or to curate labeling functions. REGAL (Rule-Enhanced Generative Active Learning) is an improved framework for weakly supervised text classification that performs active learning over labeling functions rather than individual instances. REGAL interactively creates high-quality labeling patterns from raw text, enabling a single annotator to accurately label an entire dataset after initialization with three keywords for each class. Experiments demonstrate that REGAL extracts up to 3 times as many high-accuracy labeling functions from text as current state-of-the-art methods for interactive weak supervision, enabling REGAL to dramatically reduce the annotation burden of writing labeling functions for weak supervision. Statistical analysis reveals REGAL performs equal or significantly better than interactive weak supervision for five of six commonly used natural language processing (NLP) baseline datasets.

## 1. Introduction

Collecting training labels is a necessary, fundamental hurdle in creating any supervised machine learning system. The cost of curating labels, however, can be very costly. Training a robust deep learning model generally requires on the order of 10,000+ training examples [1,2]. Recently, advances in unsupervised pretraining [3–5] have created expressive, publicly available models with smaller requirements for task adaptation via fine tuning. Pretrained models in specific domains (e.g., clinical, biomedical, legal), refs. [6,7] have extended benefits to new domains.

Researchers have improved automated text class labeling using solutions that include active learning [8], domain adaptation of pretrained models, self-training on confident model predictions [9–11], noisy supervision using labeling heuristics [12], and crowd-sourced labeling [13].

Active learning [14] seeks to reduce the labeling burden by initializing a model with a very small set of seed labels, then iteratively solicits batches of labels on “highly informative” unlabeled instances. Active learning allows a model to be robustly trained on a small subset of data while attaining performance similar to a model trained on a much larger dataset. While active learning provides large gains compared to random instance labeling, significant work is still required to label individual data instances.

Weak supervision provides multiple overlapping supervision sources in the form of independent labeling rules, then probabilistically disambiguates sources to obtain predictions. Since a single labeling rule (also called a labeling function) can label a large proportion of a dataset, a small number of labeling rules can lead to significant gains in efficiency while minimizing annotation efforts. The main difficulty in weak supervision is the need to curate these labeling functions, which can be deceptively complex and nuanced.

**Figure 1.**
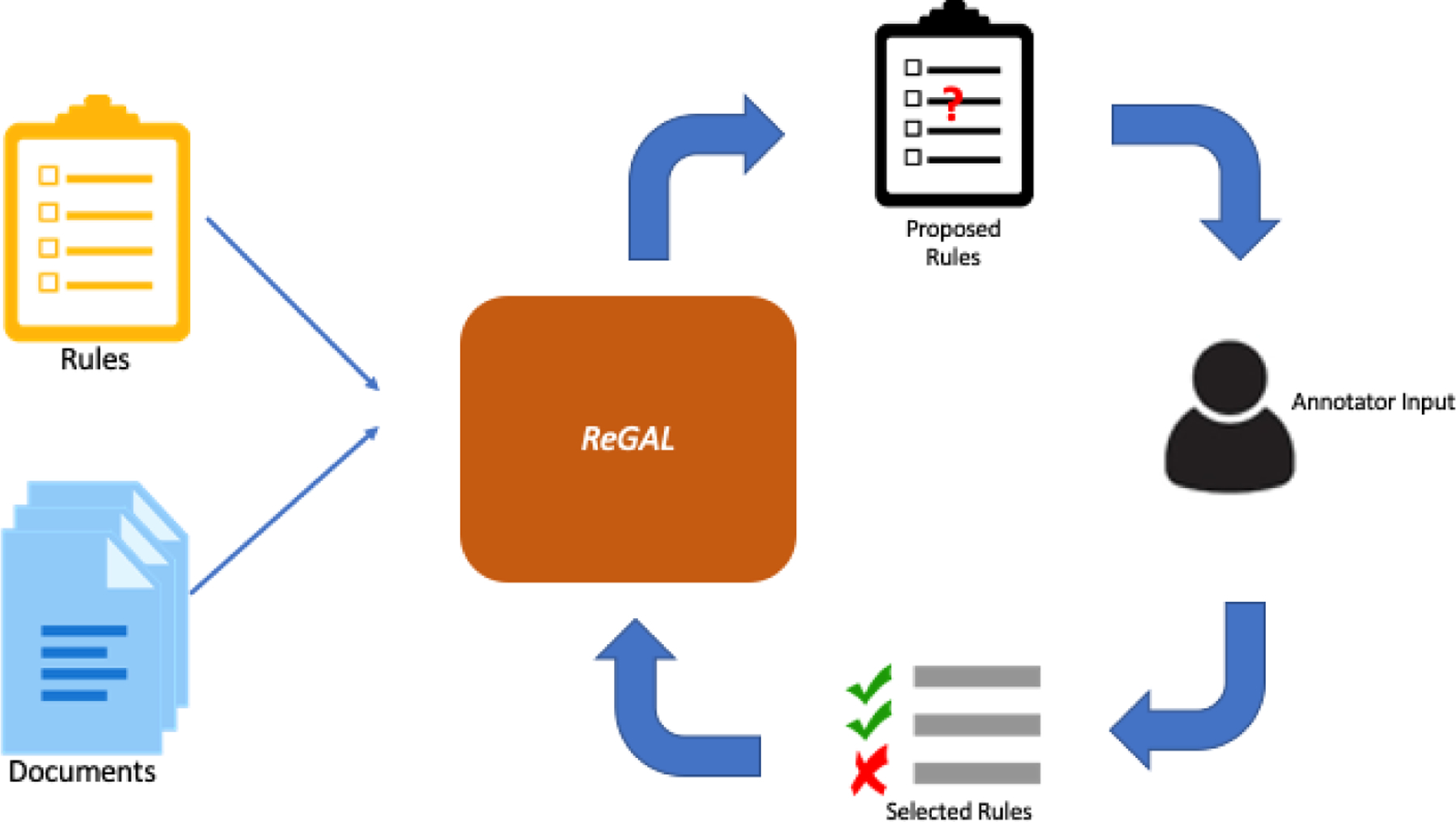
REGAL model setup. REGAL takes unlabeled documents and seed rules as input. It then iteratively proposes new labeling functions by extracting high-quality patterns from the training data and soliciting user feedback about which to keep.

To address limitations of prior labeling methods, we synthesize ideas from active learning, pretraining, and weak supervision to create REGAL, which performs active learning over model-generated labeling functions. REGAL accelerates data labeling by interactively soliciting human feedback on labeling functions instead of individual data points. It accomplishes this by (1) extracting high-confidence labeling rules from input documents, (2) soliciting labels on these proposed rules from a human user, and (3) denoising overlap between chosen labeling functions to create high-confidence labels. This framework, depicted in Figure 1 enables REGAL to seek feedback on areas of model weakness while simultaneously labeling large swaths of examples.

## 2. Preliminaries

**Figure 2.**
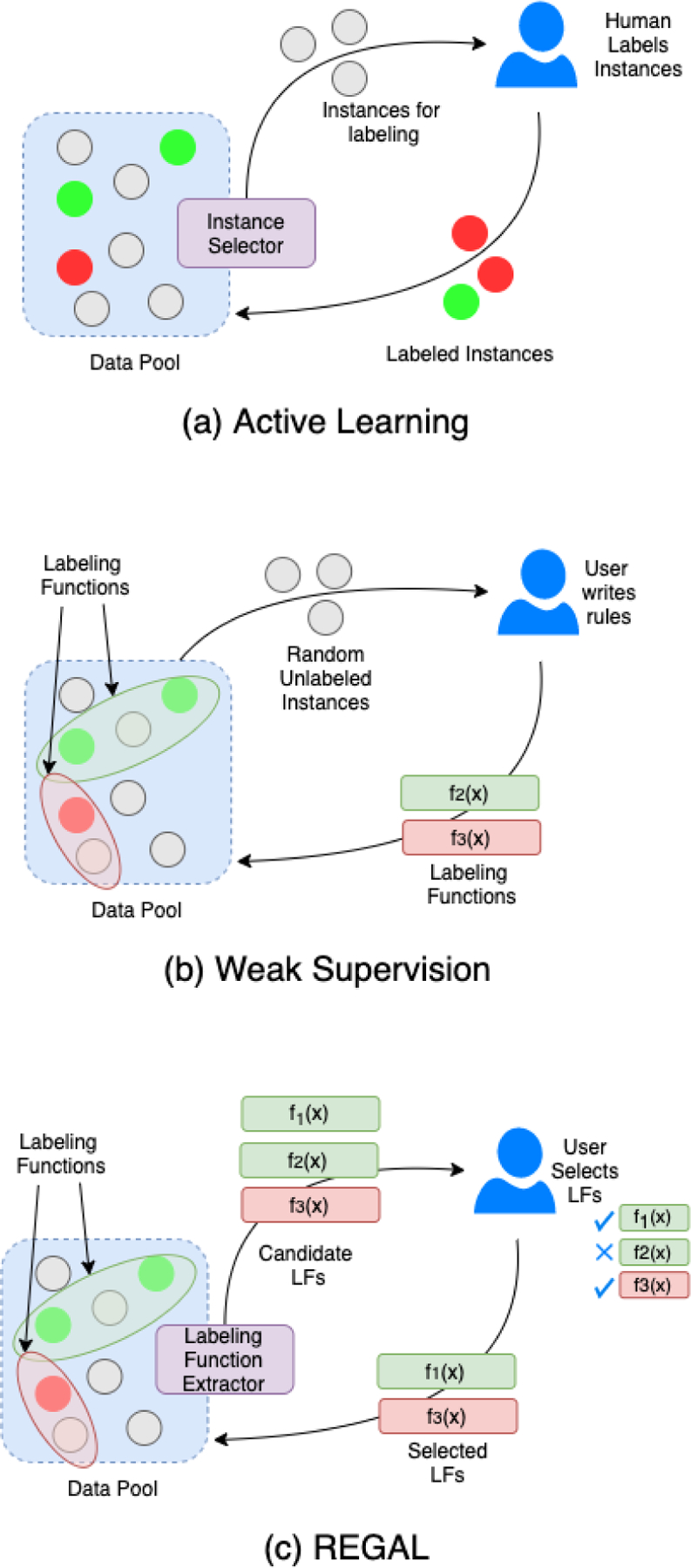
Labeling structure for traditional active learning, weak supervision, and REGAL. In traditional active learning, high-value instances are selected and sent to a human annotators for labeling. In traditional weak supervision, annotators write rules based on patterns they observe in data. REGAL synthesizes these two approaches by extracting high-value candidate LFs which are then filtered by human annotators.

REGAL proposes multiple, high-quality sources of weak supervision to improve labeling on a source dataset as formally defined below. Figure 2 illustrates the differences between active learning, weak supervision, and REGAL.

### 2.1. Problem Formulation

It is assumed that for a given a set of documents $D=\left\{d_{1},d_{2},\ldots ,d_{\left|D\right|}\right\}$, each of which has a (possibly unknown) classification label $c_{i}\in C$. Each document *d*_*i*_ = [*v*_*i*,1_, *v*_*i*,2_, …, *v*_*i*,*T*_] represents a sequence of tokens from the vocabulary V, where tokens drawn from V could be words, subwords, characters, etc.

It is assumed there is no access to ground-truth labels for the documents in the training set. However, there are a small number of heuristic labeling functions (LFs) given which provide limited initial supervision for each class. $R=\left\{r_{1},r_{2},\ldots ,r_{l}\right\}$, where each $r_{j}:D\to C\cup \left\{C_{\text{abstain}}\right\}$ is a function that maps documents to a class label in C or abstains from labeling. This set of LFs induces a vector of noisy labels for each document, denoted *£*_*i*_ = [*r*_1_(*d*_*i*_), *r*_2_(*d*_*i*_), …, *r*_*l*_(*d*_*i*_)]^*T*^. Because LFs act as rule-based labelers, we freely interchange the terms “labeling function” and “rule” throughout the paper.

### 2.2. Challenges

Weakly supervised text classification presents three main challenges: label noise, label incompleteness, and annotator effort. For a lengthier discussion of different sources of label noise and the different types of algorithms used to address label incompleteness, see [15].

#### 2.2.1. Label Noise

Label noise is the problem of labeling functions generating incorrect labels for particular data instances. This problem generally occurs when a specified labeling function is too general and, thus, mislabels instances into the wrong class. The diversity of language presents an extremely large space of possible misapplications for a single labeling function and enumerating these misapplications can be prohibitively expensive.

Recent works to address label noise include: generative label denoising [12,16], self-training on synthetic examples generated with latent variable models [17], using a neural network to identify improper applications of labeling functions using labeled rule exemplars [18], and active learning on instances with conflicting labels [19]. REGAL seeks to reduce label noise by automatically learning rules designed to differentiate between separate classes.

#### 2.2.2. Label Incompleteness

Label incompleteness is the insufficiency of labeling functions to assign labels to particular slices of a dataset. It occurs when the syntactic and semantic patterns in a subset of examples do not lie within the scope of the given labeling functions. Label incompleteness is particularly pervasive in the long tails of a dataset, which often contain more diverse, difficult instances. For this reason, label incompleteness commonly manifests in low-resource or highly technical domains where differences in nomenclature lead to large labeling gaps.

Approaches to tackle label incompletness include differentiable soft-matching of labeling rules to unlabeled instances [20], automatic rule generation using pre-specified rule patterns [21,22], co-training a rule-based labeling module with a deep learning module capable of matching unlabeled instances [11,17], and encouraging LF diversity by interactively soliciting LFs for unlabeled instances [19].

#### 2.2.3. Annotator Effort

Many domains require subject matter experts (SMEs) to annotate correctly. However, SMEs have cost and time constraints. These constraints are often most pressing in domains requiring the most expertise (e.g., biomedical), which is precisely where expert input is most valuable. By presenting annotators with candidate labeling rules, REGAL reduces the time necessary to specify rules by hand, thereby increasing annotator efficiency.

### 2.3. Objectives

REGAL is a model that interactively generates labeling functions from a text corpus with a small set of sparse, noisy labels. REGAL addresses text labeling challenges by automatically proposing labeling rules designed to (1) disambiguate instances with conflicting LF-induced labels and (2) extend coverage to unlabeled portions of the dataset. As annotators generate labels, REGAL can adapt to new labeling needs as the set of labels expands.

## 3. Methods

**Figure 3.**
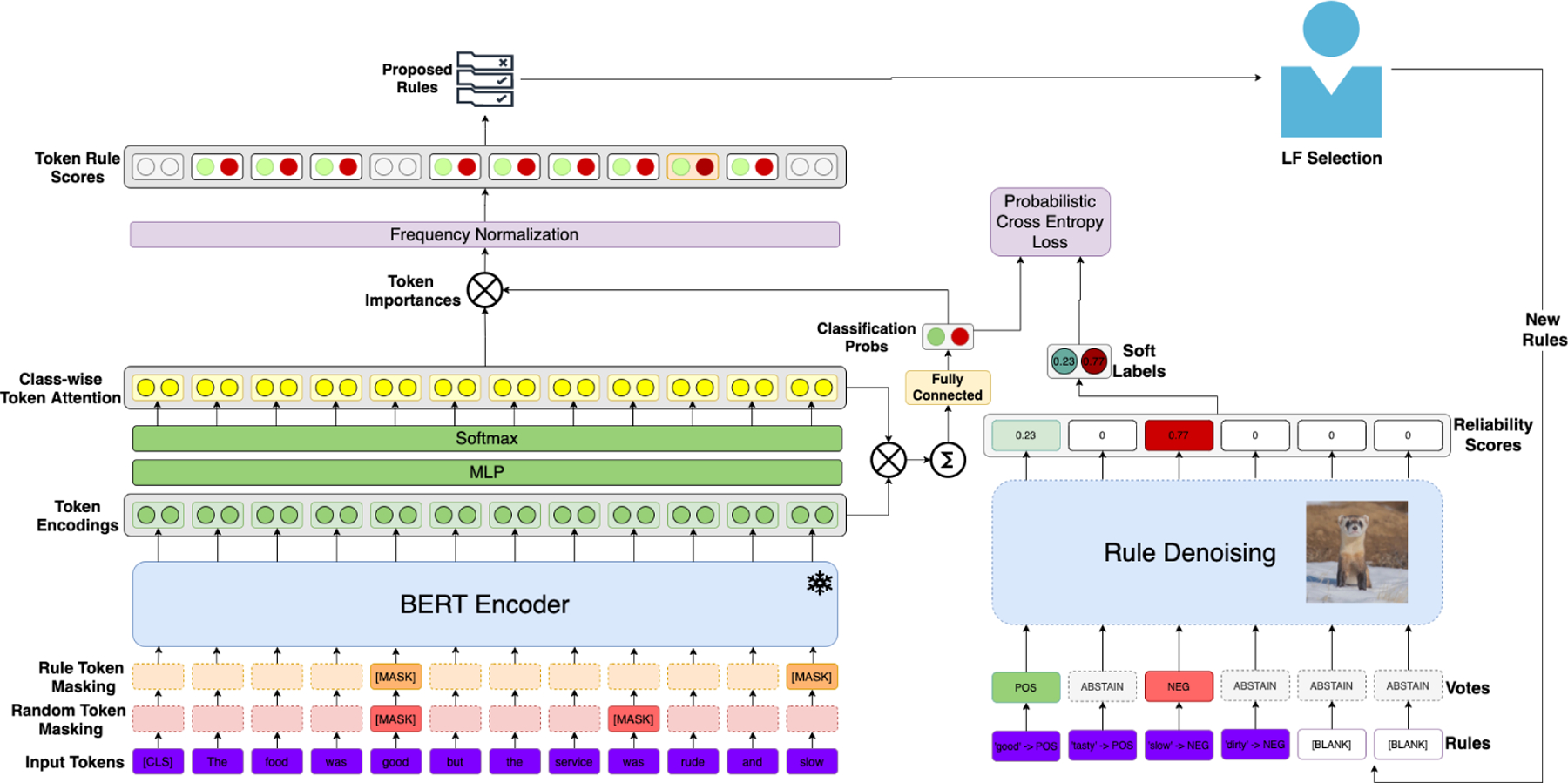
Model architecture for REGAL.

REGAL’s architecture is shown in Figure 3. REGAL is composed of four components:

1. TextEncoder: Encodes semantically meaningful information about each token and document into contextualized token embeddings.
2. SnippetSelector: Extracts relevant phrases for document classification.
3. RuleProposer: Generates candidate labeling functions from extracted snippets.
4. RuleDenoiser: Produces probabilistic labels for all documents using labeling functions and document embeddings.

### 3.1. Text Encoder

REGAL begins with a TextEncoder module whose purpose is to create semantically meaningful embeddings for each document and its individual tokens. Token embeddings are used by the snippet selector and rule proposer to construct meaningful rules. The document embeddings are used by the rule denoiser to weight LF relevance for individual instances.

We create TextEncoder as a bidirectional, transformer-based encoder [23] using a pretrained, uncased BERT-base model [3] provided by Huggingface [24]. It allows each token’s embedding to be conditioned on all other input text in the document, allowing them to capture rich contextual information. We use the outputs *h*_*i*,*t*_ of the last layer as token embeddings:

(1) $$\left[h_{i,1},\ldots ,h_{i,T}\right]=enc\left(\left[v_{i,1},\ldots ,v_{i,T}\right]\right)$$

We will henceforth let *H*_*i*_ = [**h**_*i*,1_, …, **h**_*i*,*T*_] represent the sequence token embeddings from document *d*_*i*_.

In addition to initializing TextEncoder with a BERT-base, we encourage the encoder to further learn contextual information about labeling rules using a masked language modeling (MLM) objective. Our masking budget consists of all of tokens used in LFs as well as a random 10% of tokens from the sequence. Each token is either masked or noised according to the strategy in Devlin et al. [3], and TextEncoder is required to predict the correct token in each case. Thus, TextEncoder continually learns new labeling cues rather than memorizing simple labeling functions. Optimization is performed using cross entropy loss over the masked/noised tokens, denoted as $L_{MLM}$.

### 3.2. Snippet Selector

After producing expressive token embeddings, those most useful for creating labeling rules must be selected. Accordingly, we develop a SnippetSelector module to identify which pieces of text are most useful for developing precise labeling functions and rich document representations.

SnippetSelector learns to extract words and phrases that are indicative of an individual class label. A classwise attention mechanism over tokens identifies and extracts the token and document level information necessary to generate expressive, class-specific labeling functions. SnippetSelector also calculates each document’s probability of belonging to each class. These probabilities serve as suitability scores as to how well-equipped a document is to generate LF keywords of that class.

SnippetSelector takes as inputs the token embeddings from the document encoder and produces class-specific token attention $a_{i,t}^{\left(c\right)}$, document embeddings **e**_*i*_, and document-level class probabilities $p_{i}=\left[p_{i}^{\left(1\right)},\ldots ,p_{i}^{\left(C\right)}\right]$, which are computed as follows.

First, class-specific attention scores are calculated for each token in our document. Class-specific attention scores are used by the rule proposal network to generate new labeling rules and are calculated as follows:

(2) $$a_{i,t}=W_{2}^{a}\text{tanh}\left(W_{1}^{a}H_{i}\right)$$

where $W_{1}^{a}\in R^{m_{2}\times m_{1}}$ and $W_{2}^{a}\in R^{c\times m_{2}}$. These scores are then used to calculate a class-specific document representation

(3) $${\tilde{e}}_{i}^{\left(c\right)}=\sum_{t=1}^{T}a_{i,t}^{\left(c\right)}h_{i,t}$$

These are in turn aggregated into an overall document representation with class weights *η*_*c*_

(4) $$e_{i}=\sum_{c=1}^{C}\eta_{c}{\tilde{e}}_{i}^{\left(c\right)}$$

This representation will be used by the rule attention submodule to estimate conditional LF reliability.

The class-specific embeddings ${\tilde{e}}_{i}^{\left(c\right)}$ are also used to compute the document’s class probabilities:

(5) $$p_{i}=\text{softmax}\;\left(\left[{\hat{p}}_{i}^{\left(1\right)},\ldots ,{\hat{p}}_{i}^{\left(C\right)}\right]\right)$$

where $p_{i}^{\left(c\right)}=w_{p}^{{\left(c\right)}^{T}}{\tilde{e}}_{i}^{\left(c\right)}$ and $W_{p}^{\left(c\right)}$ is a weight vector corresponding to each class. In addition to serving as this submodule’s prediction of the document’s label, these probabilities also serve as measures of the document’s suitability to contribute to LFs of each particular class.

Because BERT tokens are wordpiece subword units, the SnippetSelector aggregates subword attentions to a word level by simply summing all of the subword attentions that correspond to a particular word. These are further aggregated into phrase weights by summing over all words in a phrase. Phrase attentions are then passed to the rule proposal network to create rules that are displayed to users for adjudication.

### 3.3. Rule Proposal Network

REGAL’s RuleProposer module REGAL to measure the quality of keyword and phrase based rules given a set of seed rules. This can be easily extended to create rules from a set of seed labels as well. The RuleProposer takes as inputs both the class-conditioned word level attention $a_{i,t}^{c}$ and document-level class probabilities **p**_*i*_ and outputs a score $\tau_{j}^{\left(c\right)}$ for each $v_{j}\in V$ corresponding to how strongly *v*_*j*_ represents class *c*. These scores are calculated as:

(6) $$\tau_{j}^{\left(c\right)}c=\frac{1}{{\left|v_{j}\right|}^{\gamma }}\sum_{i=1}^{\left|D\right|}\sum_{t=1}^{T}1_{v_{i,t}=v_{j}}p_{i}^{\left(c\right)}a_{i,t}^{\left(c\right)}$$

Here, *γ* ∈ [0, 1] is a parameter that controls how much REGAL’s RuleProposer balances between the coverage of a phrase (i.e., how often it occurs) and its instance level importance. Low values of *γ* favor phrases with high coverage while high values of *γ* favor LFs based on highly precise phrases with less regard for coverage. Since the types of rules needed may differ as training progresses, we allow users to choose *γ* for each round of proposed rules. In practice, we find that *γ* ∈ [0.5, 0.9] tend to produce good rules.

Once candidate rules have been generated, they are passed through a PhrasePruner module that filters them to improve coverage and discriminative capacity. The PhrasePruner performs two pruning steps. First, it trims rules below a certain count threshold *α*. Trimming ensures that chosen rules have sufficient coverage to be useful. Second, we perform polarity pruning, which limits candidate phrases to those that a difference of at least *β* between the first and second highest scoring classes. Polarity pruning ensures that rules are highly specific to a single class and eliminates phrases containing stopwords, punctuation, and other tokens not particularly relevant to distinguishing classes. Scores for all but the highest scoring class are set to 0 to avoid any phrase being chosen as a representative of multiple classes. In practice, we find that *α* >= 10 and *β* = 0.4/|*C*| tend to work well.

Of the remaining phrase scores $\tau_{j}^{\left(c\right)}$, the RuleProposer proposes up to *k* new LFs for each class by choosing the top *k* scoring phrases $\left\{v_{1}^{\left(c\right)},\ldots ,v_{k}^{\left(c\right)}\right\}$ for each class *c*′. These tokens each induce a labeling function of the form $\text{HAS}\left(\text{x},\;v_{i}^{\left(c\right)}\right)\to c$, where the class label *c* is assigned to a text *x* if it contains the token $v_{i}^{\left(c\right)}$.

### 3.4. Rule Denoiser

As multiple general-purpose LFs are proposed, it is inevitable that some will conflict. Accordingly, we utilize a rule denoiser developed in [25] to learn probabilistic labels based on the rules matched to each instance.

We train these soft labels and the class probabilities **p**_*i*_ from SnippetSelector using probabilistic cross entropy loss:

(7) $$L_{TOK}=-\sum_{c=1}^{c}y_{i}^{\left(c\right)}\text{log}\left(p_{i}^{\left(c\right)}\right)$$

Note that the methods in this section can easily be modified to support multilabel classification. This could be performed by using multiple label models (one for each class) and by replacing the single multi-class cross entropy loss with sum of the individual binary cross entropy loss terms for each class.

### 3.5. Model Optimization

The entire model is optimized by minimizing the unweighted sum of the loss functions of its components:

(8) $$L=L_{MLM}+L_{TOK}$$

## 4. Experiments and Discussion

### 4.1. Datasets

REGAL’s performance is evaluated on a number of sentiment and topic classification datasets:

**Yelp** is a collection of Yelp restaurant reviews classified according to their sentiment;
**IMDB** is a set of movie reviews classified according to sentiment [26];
**AGnews** is a news corpus for topic classification with four classes: sports, technology, politics, and business [27];
**BiasBios** is a set of biographies of individuals with classes based on profession. We use the binary classification subsets utilized in [28]: (1) Professor vs. Physician, (2) Professor vs. Teacher, (3) Journalist vs. Photographer, and (4) Painter vs. Architect.

Basic summary statistics on our data are found in Table 1.

### 4.2. Baseline Models

We compare REGAL’s ability to identify promising keyword LFs to the baseline models models described below.

#### 4.2.1. Snuba or Reef

Snuba [21], recently renamed Reef, is an automated method of extending the coverage of a small, labeled dataset by automatically generating a subset of labeling functions from this labeled subset. It uses an acquisition function for new LFs consisting of a weighted average of the F1 score on the dev set and the Jaccard distance of a newly proposed rule to the current LF set.

#### 4.2.2. Interactive Weak Supervision

Interactive Weak Supervision [28] is very similar to REGAL and uses active learning to evaluate rules based on the documents they match. IWS evaluates rules via an ensemble of small multi-layer perceptrons (MLPs) and prioritizes the labeling uncertain rules close to the decision boundary using the saddle acquisition function described in [29].

#### 4.2.3. Fully Supervised BERT Model (FS BERT)

A fully-supervised BERT model is used to compare the performance of the labeling models developed from REGAL’s proposed rules.

### 4.3. Training Setup

REGAL requires a user to provide at least some labeling signal to prime the rule generator. Accordingly, we provide three phrase-matching LFs for each class of each dataset. Keywords for seed rules are shown in the Appendix B. If the LF’s phrase is found in document *d*_*i*_, the LF assigns its label; otherwise, it abstains from labeling *d*_*i*_.

REGAL is run for five rounds of LF extraction with *α* = 0.7 and one epoch of training between each round of rule extraction. Each extracted phrase candidate is required to occur in at least 20 documents to be considered as a labeling function. After each round of training and accumulating rule scores, we take the solicit labels on the top *m* rules for each class, where *m* = *min*(50, *k*) and *k* is the number of rules above the polarity threshold. Solicited labels are evaluated by an oracle evaluator which accepts a proposed rule *r*_*j*_ if *accuracy*(*r*_*j*_) > *ϕ* on matched samples. We choose *ϕ* = 0.7 as our acceptance threshold. Further parameter settings for training can be found in the Appendix C.

### 4.4. Rule Extraction

REGAL’s key feature is the ability to extract expressive, high-coverage labeling rules from text. The ability of REGAL to identify promising rules based on the provided seed rules evaluated.

We compare LFs selected by REGAL to those from other methods based on their coverage and accuracy, each macro-averaged across LFs. We additionally compare how the labeling functions from different models work together to train a label denoising model to generate probabilistic labels of the data. Downstream performance is evaluated using the accuracy and area under the receiver operator characteristic curve (AUC). The results of this comparison are shown in Table 2.

From these results, we observe that REGAL consistently produces more LFs than other methods, but that the average accuracy of these is often slightly below the LFs produced by Reef and IWS. However, the average accuracy for REGAL could also be distorted if the average accuracy of its rules is lowered by the large number of additional rules not identified by IWS. To examine this, we compared the rules produced by REGAL and IWS using a Mann–Whitney–Wilcoxon [31] test. Specifically, we test the hypothesis that one produces rules that are significantly more accurate than those produced by the other. The results of these tests is given in Table 3. These tests reveal that the accuracy of rules from REGAL and IWS are very comparable, with no significant difference on four of six datasets and each method significantly outperforming the other on one dataset each.

Another interesting result is both models often see lower accuracy from downstream label models than the average accuracy of LFs input into said label models. Upon further investigation, this phenomenon appears to be occur due to imbalance in the total number of labeling votes for each individual class. To test this hypothesis, we balanced the number of noisy label votes to reflect a roughly even class balance. Balancing was performed by randomly downsampling labeling functions from dominant classes until all classes had roughly the same number of total LF votes for each class. The resultant accuracy scores before and after balancing are shown in Table 4. These results reveal that balancing LF outputs tends to increase accuracy for Snorkel label models and for majority voting, despite reducing the amount of data used for training. However, balancing tends to reduce AUC scores, implying that the additional labels do assist in rank-ordering instances even if these instances are mislabeled due to the decision boundary cutoff. Because of this skew, labels and probabilities produced by these label models should be used with care.

### 4.5. Qualitative LF Evaluation

The LFs extracted by REGAL are best understood through specific examples. This enables a user to inspect the extent to which LFs discovered by REGAL model semantically meaningful indicators for a particular domain, or if REGAL is rather targeting artifacts that are specific to the particular dataset in question. To this end, we present the first six rules generated by REGAL for each of our datasets in Table 5. We additionally provide samples of multi-word LFs discovered by REGAL in Table A1 in the Appendix B.

From the top rules selected, we see the type of textual clues REGAL catches to select rules. In Yelp reviews, it unsurprisingly catches words of praise to represent positive reviews and people seeking remediation for poor experiences for negative reviews. Additionally, REGAL selects many specific food entreés as positive LFs keywords, highlighting that positive reviews tend to discuss the individual food that people ordered more than negative ones. In contrast, negative LFs tend to focus on experiences outside of dining, such are retail and lodging.

Similar trends emerge in LFs selected for the professor/physician dataset. ‘Professor’ LFs tend to correspond to academic disciplines, whereas ‘physician’ LFs relate to aspects of medical practice (such as specialization or insurance) of the specific location where a physician practiced. Notably, the locations selected as rules for the physician class are lesser-known, avoiding towns with major universities that may conflict with the professor class.

Note that all of the rules selected were confirmed by oracle evaluation. This implies that REGAL selects some LFs that are data artifacts that correlate closely with one class but are not intuitive to a human annotator. In this sense, REGAL can be a useful tool for identifying artifacts that could impede the generalization of a model and be used to make models more robust.

## 5. Related Work

REGAL builds on dual foundations, active and weakly supervised learning, for text classification.

### 5.1. Active Learning

REGAL shares a few goals with active learning. First, REGAL iteratively solicits user feedback to train a robust downstream model with minimal annotation effort. Methods to perform active learning include selecting a diverse, representative set of instances to annotate [32,33], selecting the instances about which the model is least confident [29,34], and selecting the instances with the highest expected gradient norm and thus the highest expected model change [35]. Second, REGAL shares active learning’s goal to interactively solicit an optimal set of labels. However, REGAL differs by soliciting labels for labeling functions rather than individual data points. Soliciting labels for label functions increase coverage for a much larger number of instances per given label. It also enables LFs to be inductively applied to additional data not seen during training.

### 5.2. Weakly Supervised Learning

Weakly supervised learning dates back to early efforts to model the confidence of crowd-sourced labels based on inter-annotator agreement [13]. Works such as Snorkel [12,25] have adapted these ideas to learn label confidence based on the aggregation of large numbers of noisy, heuristic LFs. Weak supervision has been shown to be effective at a host of tasks, including named entity recognition [36,37], seizure detection [38], image segmentation [39], relation extraction [20], and text classification [9,11,18,40]. However, all of these models require users to define labeling functions manually, creating a usability barrier to subject matter experts not used to writing code. Some also require additional labeled instances for self-training [18,40], which REGAL does not. Recent works have reduced the barrier to scaling weak supervision by propagating labels to nearby matched examples in latent space [41] and soft-matching LFs to samples not explicitly labeled by the LF [20]. Additional studies have shown that convergence to a final set of diverse LFs can be accelerated by prompting users with high-priority examples such as those which are unlabeled or have conflicting LFs [19].

Snuba/Reef [21] uses similar weak supervision. Snuba/Reef generates LFs from a small labeled set of data and iteratively creates a diverse set of by adding new LFs using an acquisition (*r*_*k*_) = *w* * *f*_*score*_ + (1 − *w*) * *j*_*score*_, where *f*_*score*_ is the F1 score of the rule on the labeled dev set, *j*_*score*_ is the Jaccard similarity of the rule to the currently labeled set, and *w* ∈ [0, 1] a weight parameter. Snuba differs from our method in that it requires labeled data in order to generate labeling functions and it does provide a means of interactive human feedback for LF selection.

### 5.3. Combined Active Learning with Interactive Weak Supervision

REGAL is the second known work to combine active learning with interactive weak supervision for text classification using LFs. IWS [28] also enables interactive weak supervision via active learning on labeling functions. Similar to REGAL, IWS begins by enumerating all labeling functions from a particular “LF family,” such as all of the n-grams in a document. It featurizes LFs using the SVD of their matched documents, then uses an ensemble of small neural networks to estimate the accuracy of each LF. IWS then treats selecting useful LFs as an active level set estimation problem, using the saddle acquisition function Bryan et al. [29]. IWS is similar to REGAL in that both interactively select n-gram LFs via human feedback.

REGAL differs from IWS in two main areas. First, REGAL seeks attention on embeddings from pretrained language models to optimally select quality n-gram LFs, whereas IWS uses an ensemble of weak classifiers to estimate a distribution of LF quality. Second, REGAL uses a different acquisition function than IWS. REGAL seeks to maximize a combination of coverage and accuracy of proposed LFs (i.e., optimizing LF quality), whereas IWS seeks to find LFs near the decision boundary about which it is uncertain.

## 6. Conclusions and Future Work

REGAL interactively creates high-quality labeling patterns from raw text, enabling an annotator to more quickly and effectively label a data set. REGAL improves upon the challenges of label noise, label incompleteness, and annotator effort. Results confirm the combination of weak supervision with active learning provides strong performance that accelerates advancements in low-resource NLP domains by assisting human subject matter experts in labeling their text data.

Future work to improve REGAL and other interactive weak supervision methods will need to improve rule denoising and LF generation. While REGAL can identify useful labeling rules, these rules often result in unbalanced labels that skew training and overpower denoising methods meant to synthesize them. Better denoising algorithms are needed to be able to deal with this imbalance, which will also improve the performance of models such as REGAL that interact with these probabilistic labels. Given that most label models expect LFs to be fully specified before training, future work that identifies fast ways to update models with the addition of new LFs would be particularly useful. Additional work could also explore ways to generate and extract labeling functions from other, more expressive families such as regular expressions to create more precise LFs or automatically refine existing ones. More expressive labeling functions could also support sequence tagging tasks such as named entity recognition, e.g., in [36].

## Figures and Tables

**Table 1.** Summary of REGAL data and rule generation parameters. The data below describe the respective sizes of traditional train, validate, and test sets, though REGAL only extracts rules from the train set. Coverage denotes the total coverage of the initial set of seed rules, whereas Bal. Coverage denotes the accuracy after downsampling to balance class-wise labeling propensities.

Dataset	# Train	# Valid	# Test	# Classes	Coverage	Bal. Coverage
Yelp	30,400	3800	3800	2	0.2239	0.1042
IMDB	24,500	500	25,000	2	0.1798	0.1663
AG News	96,000	12,000	12,000	4	0.0963	0.0144
Journalist/Photographer	15,629	500	16,129	2	0.3211	0.2364
Professor/Physician	26,738	500	27,238	2	0.5149	0.3772
Professor/Teacher	11,794	500	12,294	2	0.5195	0.3574
Painter/Architect	5618	500	6118	2	0.4516	0.2650

**Table 2.** Performance comparison of LF extraction methods. LF accuracy and coverage are averaged over all LFs produced by the model.

Dataset	Model	# LFs	LF Acc	Coverage	LM Acc	LM AUC
AG News	IWS	_-_	_-_	_-_	_-_	_-_
	REEF/Snuba	-	-	-	-	-
	REGAL	280	0.912	0.007	0.856	-
	FS BERT *	-	-	-	0.952	-
IMDB	IWS	35	**0.807**	0.065	**0.811**	**0.883**
	REEF/Snuba	50	0.729	**0.068**	0.722	0.787
	REGAL	**193**	0.787	0.017	0.510	0.757
	FS BERT	-	-	-	0.914	0.974
Journalist/Photographer	IWS	110	0.877	0.033	0.898	**0.958**
	REEF/Snuba	23	**0.894**	**0.142**	**0.910**	0.944
	REGAL	**265**	0.840	0.030	0.733	0.890
	FS BERT	-	-	-	0.954	0.990
Painter/Architect	IWS	157	0.883	0.032	0.893	0.966
	REEF/Snuba	23	**0.893**	**0.140**	0.874	0.947
	REGAL	**373**	0.876	0.034	**0.897**	**0.977**
	FS BERT	-	-	-	0.968	0.995
Professor/Physician	IWS	238	0.860	0.042	**0.892**	**0.957**
	REEF/Snuba	26	**0.917**	**0.184**	0.882	0.935
	REGAL	**249**	0.876	0.041	0.794	0.871
	FS BERT	-	-	-	0.951	0.994
Professor/Teacher	IWS	**218**	0.785	0.030	0.760	**0.928**
	REEF/Snuba	12	0.562	**0.619**	0.782	0.839
	REGAL	211	**0.824**	0.029	**0.813**	0.877
	FS BERT	-	-	-	0.938	0.982
Yelp	IWS	87	0.799	0.047	0.747	0.830
	REEF/Snuba	38	**0.833**	**0.071**	**0.830**	**0.887**
	REGAL	**155**	0.803	0.018	0.770	0.837
	FS BERT	-	-	-	0.960	0.992
macro-average	IWS	140.833	**0.835**	0.041	**0.833**	**0.920**
	REEF/Snuba	28.667	0.805	**0.204**	**0.833**	0.890
	REGAL **	**241**	0.834	0.028	0.753	0.868
	FS BERT **	-	-	-	0.9475	0.988

# LFs denotes the total number of LFs selected/predicted by the model, not the number proposed. LM Acc and LM AUC represent the accuracy and area under the ROC curve, respectively, of the probabilistic labels produced by a Snorkel label model. For fully-supervised BERT models (denoted by FS BERT), accuracy and AUC are not computed with a label model.

* FS BERT results for AG News taken from [30].

** For fair comparison with IWS and REEF/Snuba, REGAL and FS BERT macro averages exclude AG News.

**Table 3.** Statistical comparison of REGAL and IWS using the Mann–Whitney–Wilcoxon (MWW) test. The methods show no significant difference except on the Journalist/Photographer and Professor/Physician datasets. After Bonferroni correction, MWW shows that REGAL outperforms IWS on Professor/Physician and IWS is outperforms REGAL on Journalist/Photographer.

Dataset	Higher Med. Acc.	MWW *p*-val.
Yelp	REGAL	0.3438
IMDB	IWS	0.1926
Journalist/Photographer	**IWS** *	**0.0066** *
Professor/Teacher	REGAL	0.2086
Professor/Physician	**REGAL** **	**0.0010** **
Painter/Architect	IWS	0.1438

* Significant at *p* < 0.05 after Bonferroni correction;

** significant at *p* < 0.01 after Bonferroni correction.

**Table 4.** Effects of balancing data on model label model performance. We balanced data by calculating the total number of noisy label votes for each class and randomly replacing votes for dominant classes until all label distribution was approximately balanced. We measure change in total coverage as well as Accuracy and AUC for both Snorkel label models and a simple majority voting LF aggregator (denoted “MV”). Imbalance Ratio reflects the ratio of most labeled class: least labeled class. Note that rows with higher imbalance ratio have tend to see larger improvements in accuracy after balancing.

Dataset	Model	Δ Accuracy	Δ AUC	MV Acc	Δ MV AUC	Δ Coverage	Imbalance Ratio
**AG News**	**REGAL**	0.011	–	−0.034	–	−0.154	2.245
**IMDB**	**IWS**	−0.002	−0.014	0.008	0.001	−0.107	1.896
	**REEF/Snuba**	0.002	0.000	0.000	0.000	−0.002	1.053
	**REGAL**	0.066	−0.068	0.083	−0.008	−0.165	3.573
**Journalist/Photographer**	**IWS**	−0.001	−0.013	−0.012	0.001	−0.112	2.492
	**REEF/Snuba**	−0.003	−0.004	−0.004	0.000	−0.006	1.493
	**REGAL**	−0.014	0.004	0.025	−0.012	−0.001	1.319
**Painter/Architect**	**IWS**	0.033	−0.014	0.022	0.007	−0.136	3.969
	**REEF/Snuba**	0.001	0.000	−0.003	−0.003	−0.004	1.340
	**REGAL**	−0.011	−0.006	0.015	−0.004	−0.001	1.238
**Professor/Physician**	**IWS**	−0.010	−0.008	0.006	−0.001	−0.002	1.170
	**REEF/Snuba**	−0.004	0.001	−0.007	−0.002	0.000	1.499
	**REGAL**	−0.026	−0.024	0.024	−0.009	0.000	1.380
**Professor/Teacher**	**IWS**	0.120	−0.033	0.146	0.075	−0.253	7.109
	**REEF/Snuba**	0.008	0.000	0.000	−0.008	0.000	1.012
	**REGAL**	−0.001	−0.013	0.000	−0.003	0.000	1.121
**Yelp**	**IWS**	0.085	0.061	0.060	−0.007	−0.140	3.285
	**REEF/Snuba**	0.003	0.002	0.001	0.000	−0.008	1.226
	**REGAL**	0.010	0.012	0.021	−0.019	−0.036	1.642

**Table 5.** Top 6 unigram labeling functions from first 5 iterations of REGAL. In some cases, REGAL did not identify LFs for particular classes at some iterations, denoted by “-”.

Dataset	Class	Iter. 1	Iter. 2	Iter. 3
**AG News**	**Sports**	‘ioc’, ‘olympic’, ‘knicks’, ‘nba’, ‘ncaa’, ‘medal’	‘mls’, ‘mvp’, ‘fc’, ‘sport’, ‘cowboys’, ‘golf’	‘102’, ‘35th’, ‘vs’, ‘2012’, ‘700th’, ‘ruud’
	**Science/Tech**	‘microprocessors’, ‘microprocessor’, ‘antivirus’, ‘workstations’, ‘passwords’, ‘mainframe’	‘xp’, ‘os’, ‘x86’, ‘sp2’, ‘worms’, ‘worm’	‘hd’, ‘666666’, ‘src’, ‘sd’, ‘br’, ‘200301151450’
	**Politics**	‘allawi’, ‘prime’, ‘ayad’, ‘iyad’, ‘kofi’, ‘sadr’	‘plo’, ‘holy’, ‘roh’, ‘troops’, ‘troop’, ‘mp’	-
	**Business**	‘futures’, ‘indexes’, ‘trading’, ‘investors’, ‘traders’, ‘shares’	‘http’, ‘www’, ‘output’, ‘bp’, ‘dow’, ‘bhp’	‘ob’
**IMDB**	**Positive**	‘enchanting’, ‘errol’, ‘astaire’, ‘matthau’, ‘witherspoon’, ‘mclaglen’	‘garcia’, ‘ruby’, ‘1939’, ‘emily’, ‘myrna’, ‘poem’	‘delight’, ‘stellar’, ‘vivid’, ‘voight’, ‘burns’, ‘dandy’
	**Negative**	‘dumbest’, ‘manos’, ‘lame’, ‘whiny’, ‘laughable’, ‘camcorder’	‘pointless’, ‘inept’, ‘inane’, ‘implausible’, ‘abysmal’, ‘cheap’	‘vomit’, ‘joke’, ‘morons’, ‘ugh’, ‘snakes’, ‘avoid’
**Journalist/Photographer**	**Photographer**	‘35mm’, ‘shoots’, ‘polaroid’, ‘head- shots’, ‘captures’, ‘portraiture’	‘exposures’, ‘kodak’, ‘nudes’, ‘viewer’, ‘imagery’, ‘colors’	‘shadows’, ‘macro’, ‘canvas’, ‘skill’, ‘poses’, ‘hobby’
	**Journalist**	‘corruption’, ‘government’, ‘cnn’, ‘previously’, ‘policy’, ‘stints’	‘governance’, ‘anchor’, ‘pbs’, ‘npr’, ‘democracy’, ‘bureau’	‘arabic’, ‘programme’, ‘elsewhere’, ‘economy’, ‘crisis’, ‘prior’
**Painter/Architect**	**Painter**	‘galleries’, ‘collections’, ‘residencies’, ‘acrylic’, ‘plein’, ‘pastels’	‘impressionist’, ‘textures’, ‘strokes’, ‘flowers’, ‘figurative’, ‘brush’	‘palette’, ‘feelings’, ‘realism’, ‘emotion’, ‘realistic’, ‘filled’
	**Architect**	‘soa’, ‘enterprise’, ‘bim’, ‘server’, ‘scalable’, ‘solutions’	‘infrastructure’, ‘methodologies’, ‘certifications’, ‘intelligence’, ‘teams’, ‘developer’	‘automation’, ‘computing’, ‘delivery’, ‘healthcare’, ‘initiatives’, ‘processing’
**Professor/Physician**	**Professor**	‘banking’, ‘democratization’, ‘verification’, ‘cooperation’, ‘governance’, ‘b’	‘security’, ‘finance’, ‘macroeconomics’, ‘microeconomics’, ‘political’, ‘law’	‘acm’, ‘optimization’, ‘mechanical’, ‘metaphysics’, ‘computational’, ‘visualization’
	**Physician**	‘specializes’, ‘alaska’, ‘takes’, ‘accepts’, ‘norfolk’, ‘ky’	‘speaks’, ‘aurora’, ‘carolinas’, ‘menorah’, ‘novant’, ‘affiliated’	‘vidant’, ‘anthonys’, ‘southside’, ‘fluent’, ‘hindi’, ‘osf’
**Professor/Teacher**	**Teacher**	‘grades’, ‘ages’, ‘eighth’, ‘aged’, ‘graders’, ‘grade’	‘ratings’, ‘sixth’, ‘fifth’, ‘fun’, ‘fourth’, ‘tutoring’	‘pupils’, ‘favorite’, ‘cooking’, ‘volunteering’, ‘comparing’, ‘friends’
	**Professor**	‘governance’, ‘constitutional’, ‘cooperation’, ‘regulation’, ‘democracy’, ‘finance’	‘econometrics’, ‘banking’, ‘economy’, ‘markets’, ‘entrepreneurship’, ‘economic’	‘globalization’, ‘optimization’, ‘firms’, ‘statistical’, ‘conflict’, ‘tax’
**Yelp**	**Positive**	‘phenomenal’, ‘yummy’, ‘delectable’, ‘favorite’, ‘amazing’, ‘atmosphere’	‘terrific’, ‘heavenly’, ‘notch’, ‘hearty’, ‘chic’, ‘stylish’	‘handmade’, ‘kale’, ‘cozy’, ‘carpaccio’, ‘tender’, ‘fave’
	**Negative**	‘refund’, ‘pharmacy’, ‘disrespectful’, ‘refunded’, ‘warranty’, ‘rudest’	‘cancel’, ‘scam’, ‘confirmed’, ‘dealership’, ‘driver’, ‘appt’	‘receipt’, ‘confirm’, ‘reply’, ‘cox’, ‘clerk’, ‘policy’

## Data Availability

Access to download the REGAL code can be found on GitHub: www.github.com/pathology-dynamics/regal; accessed on 17 December 2021.

## Appendix A. Datasets and Preprocessing

For each of our datasets, we held out small validation and test sets to ensure that REGAL was training properly and to evaluate how created LFs were contributing to model performance. Validation and test sets were not used in rule selection. A summary of the statistics of each of our datasets can be found in Table 1 in the main ppaer. We also include the coverage of our initial seed rules and the size of the balanced, downsampled labeled dataset used to begin model training.

Minimal preprocessing was performed only to preserve consistency in the text. Text in datasets was processed to remove accents and special characters. All examples were truncated or padded to 128 word-piece tokens.

While stopwords and punctuation were not removed from the text, LFs with punctuation were removed, as were unigram LFs that were included as stopwords. For sentiment datasets, we converted contractions containing a negation into their non-contracted form (e.g., “didn’t” became “did not”) to insure consistency.

## Appendix B. Seed Labeling Rules

Here we describe the three seed rules used for each class for each baseline dataset.

Yelp LFs:

Positive: ‘best’, ‘excellent’, ‘awesome’
Negative: ‘worst’, ‘awful’, ‘nasty’

Professor/Physician LFs:

Professor: ‘professor’, ‘science’, ‘published’
Teacher: ‘medical’, ‘practice’, ‘physician’

Journalist/Photographer LFs:

Journalist: ‘journalism’, ‘writing’, ‘news’
Photographer: ‘photographer’, ‘studio’, ‘fashion’

Professor/Teacher LFs:

Professor: ‘professor’, ‘research’, ‘published’
Teacher: ‘elementary’, ‘children’, ‘teacher’

Painter/Architect LFs:

Painter: ‘painting’, ‘art’, ‘gallery’
Architect: ‘building’, ‘architect’, ‘residential’

IMDB LFs:

Positive: ‘masterpiece’, ‘excellent’, ‘wonderful’
Negative: ‘worst’, ‘awful’, ‘garbage’

**Table A1.** Top 6 bigram labeling functions from the first iteration of REGAL. Cases where REGAL did not select any bigram LFs are denoted by “-”.

Dataset	Class	Length 2 Rules
**AG News**	**Sports**	‘2006 world’, ‘93 -’, ‘- star’, ‘half goals’, ‘world short’, ‘1 draw’
	**Science/Tech**	‘worm that’, ‘os x’, ‘/ l’, ‘data -’, ‘a flaw’, ‘chart)’
	**Politics**	‘labour party’, ‘labor party’, ‘s party’, ‘al -’, ‘bush “, ‘pro -’
	**Business**	‘- wall’, ‘$ 46’, ‘up 0’, ‘$ 85’, ‘$ 43’, ‘a &’
**IMDB**	**Positive**	‘kelly and’, ‘claire danes’, ‘george burns’, ‘jack lemmon’, ‘michael jackson’, ‘hong kong’
	**Negative**	‘just really’, ‘plain stupid’, ‘maybe if’, ‘avoid it’, ‘so stupid’, ‘stupid the’
**Journalist/Photographer**	**Photographer**	-
	**Journalist**	‘see less’, ‘twitter :’
**Painter/Architect**	**Painter**	‘attended the’, ‘public collections’, ‘collections including’
	**Architect**	-
**Professor/Physician**	**Professor**	‘of financial’, ‘see less’, ‘film and’, ‘and society’, ‘fiction and’,’_b’
	**Physician**	‘oh and’, ‘va and’, ‘la and’, ‘tn and’, ‘ca and’, ‘ok and’
**Professor/Teacher**	**Teacher**	‘childhood education’, ‘early childhood’, ‘primary school’, ‘of 4’, ‘special education’, ‘rating at’
	**Professor**	‘modeling and’, ‘and computational’, ‘climate change’, ‘and organizational’, ‘of government’, ‘nsf career’
**Yelp**	**Positive**	‘affordable and’, ‘food good’, ‘highly recommended’, ‘highly recommend’, ‘top notch’, ‘definitely recommend’
	**Negative**	‘never again’, ‘never recommend’, ‘ever again’, ‘very bad’, ‘never going’, ‘my card’

## Appendix C. Additional Training Details

We trained REGAL for 5 epochs on the labeled subset of data using a batch size of 128 and Adam optimizer with a learning rate 0.0001. At the end of each epoch, REGAL proposed 40 rules for each class. After each epoch, we reset model weights as done in traditional active learning [8] to prevent overfitting and corresponding rule quality degradation. We additionally stopped REGAL early if, after an epoch of training, no rules with sufficient high class polarity were available to propose. Lack of rules with sufficient high class polarity indicated that performance had saturated.

During training, some label classes tend to more readily yield viable, high-coverage rules than others, which leads to imbalance in the noisy labels. This phenomenon cripples the label denoising model, which impedes model training and the learning of additional rules. We solve this problem by randomly downsampling the noisy labels during model training to contain roughly equal numbers of each class. This leads to greater stability in LF generation.

For all binary datasets, we followed the example of [11] by requiring all labeled examples used in training to be matched by at least 2 LFs during training for greater model stability.

## Appendix D. Generated Rules

In addition to the keyword rules displayed in the main text, REGAL is capable of generating n-gram rules of arbitrary length. This allows REGAL to identify more complex linguistic phenomena. N-gram rules (for *n* > 1 generally have substantially lower coverage than unigram rules and may or may not improve downstream model performance. In this section, we display a sample of 2-gram rules discovered for each dataset in Table A1. We additionally allowed our models to search for rules with more tokens, but discovered that n-gram rules for *n* ≥ 3 are generally less valuable. However, it is possible longer n-grams may be useful for other types of datasets.

## Appendix E. Mann–Whitney-Wilcoxon Test

The Mann–Whitney-Wilcoxon test [31,42] is a non-parametric alternative for comparing independent samples of two non-normally distributed random variables. It tests the null hypothesis that both samples (samples “*a*” and “*b*” o) are drawn from the same population *P*. To perform the test, first pool all samples from *a* and *b* together and rank them from 1 to *N*, where *N* = *n*_*a*_ + *n*_*b*_ is the combined sample size of the two groups. Next, divide the ranked samples into their respective sub-populations and calculate $T_{a}=\sum_{r\in a}r$, i.e., the sum of the ranks of samples in *a*. *T*_*b*_ is calculated similarly. Finally, the uniformly distributed test statistic, U, is calculated as:

(A1)
$$U=\{\begin{array}{c} T_{a}-\frac{n_{a}\left(n_{a}+1\right)}{2},\;\text{if}\;n_{a}>n_{b}\\ T_{b}-\frac{n_{b}\left(n_{b}+1\right)}{2},\;\text{otherwise} \end{array}$$

Values of the Mann–Whitney-Wilcoxon test were computed using the statistics module of the scipy python package [43]. A standard Bonferonni correction was utilized to lower the p-value threshold for significance in the case of multiple comparisons. A traditional family-wise alpha equal to 0.05 was used. The Bonferonni correction takes the family-wise alpha and divides it be the number of relevant performed comparisons for a given group of pairwise tests utilizing the same sample(s). The Bonferroni correction for multiple pair-wise comparisons is a conservative assessment of significance, as it decreases the likelihood of a Type 1 error (i.e., a false positive - rejecting the null hypothesis when it is, in fact, true).

## Abbreviations

LF: Labeling Function
LM: Label Model
AUC: Area Under the Curve
IWS: Interactive Weak Supervision
REGAL: Rule-Enhanced Generative Active Learning
